# Roco Proteins and the Parkinson’s Disease-Associated LRRK2

**DOI:** 10.3390/ijms19124074

**Published:** 2018-12-17

**Authors:** Jingling Liao, Quyen Q. Hoang

**Affiliations:** 1 Department of Public Health, Wuhan University of Science and Technology School of Medicine, Wuhan 430081, China; 2 Department of Biochemistry and Molecular Biology, Indiana University School of Medicine, Indianapolis, IN 46202, USA; 3 Department of Neurology, Indiana University School of Medicine, Indianapolis, IN 46202, USA; 4 Stark Neurosciences Research Institute, Indiana University School of Medicine, Indianapolis, IN 46202, USA

**Keywords:** Roc, Intramolecular mechanism, Small GTPase, LRRK2

## Abstract

Small G-proteins are structurally-conserved modules that function as molecular on-off switches. They function in many different cellular processes with differential specificity determined by the unique effector-binding surfaces, which undergo conformational changes during the switching action. These switches are typically standalone monomeric modules that form transient heterodimers with specific effector proteins in the ‘on’ state, and cycle to back to the monomeric conformation in the ‘off’ state. A new class of small G-proteins called “Roco” was discovered about a decade ago; this class is distinct from the typical G-proteins in several intriguing ways. Their switch module resides within a polypeptide chain of a large multi-domain protein, always adjacent to a unique domain called COR, and its effector kinase often resides within the same polypeptide. As such, the mechanisms of action of the Roco G-proteins are likely to differ from those of the typical G-proteins. Understanding these mechanisms is important because aberrant activity in the human Roco protein LRRK2 is associated with the pathogenesis of Parkinson’s disease. This review provides an update on the current state of our understanding of the Roco G-proteins and the prospects of targeting them for therapeutic purposes.

## 1. Introduction

The Ras/GTPase superfamily consists of monomeric small GTPases with molecular masses of 20–30 kDa. Small GTPases act as molecular switches that cycle between the active GTP-bound and inactive GDP-bound states. The functional cycle is regulated mainly by two different groups of regulatory proteins: (1) The Guanine nucleotide exchange factors (GEFs), which facilitate GDP dissociation from the small GTPases and exchanging with GTP leading to the transmission of various upstream signals; and (2) GTPase-activating proteins (GAPs), which accelerate the speed of the intrinsic GTP hydrolysis of the GTPase, thereby resulting in the inactivation of the molecular switch. The Ras/GTPase superfamily perform their cellular function by activating downstream protein kinase cascades. The Ras signaling pathway is a classic example in the mitogen-activated protein kinase (MAPK) pathway. 

**Figure 1:**
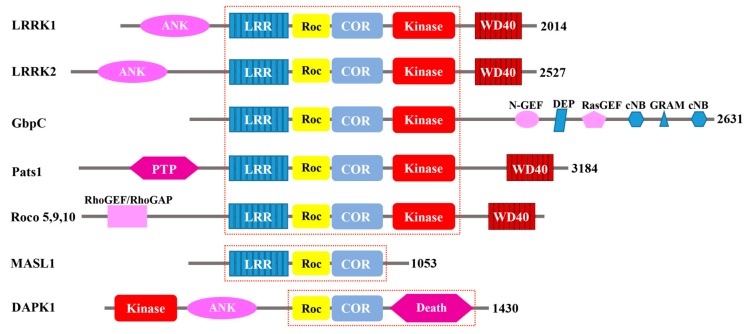
Schematic depiction of the Roco proteins. A common feature among them is the central Roc-COR domains, which are flanked by various functional domains. Ank, ankyrin domain; LRR, leucine-rich repeats; ROC, Ras of complex proteins; COR, C-terminal of Roc; Kinase, kinase domain; WD40, WD40-like beta-propeller repeat domain; cNB, cyclic nucleotide binding; DEP, Dishevelled, EGL-10, pleckstrin domain; GEF, guanine nucleotide exchange factor; GRAM, glucosyltransferases, Rab-like GTPase activators and myotubularins domain; Death, death domain; PTP, protein tyrosine phosphatase domain; RhoGEF/RhoGAP, RhoGEF/GAP domain. The red dotted-lines highlight the features that differentiate the three groups of Roco proteins.

In the past decade, a novel group of Ras/GTPase superfamily has been discovered, which is called the Ras of complex proteins (Roc). These Roc GTPase exist as a domain within a large multi-domain protein [1]. Roc was first described in the slim mold *Dictyostelium discoideum* which possesses a complex protein termed GbpC (cGMP binding protein C) [2]. P.J.M. Van Haastert’s and J.L. Smith’s group identified that GbpC contains a number of different domains, including a leucine-rich repeats domain (LRR), a Ras domain, MEK kinase domain, a Ras guanine nucleotide exchange factor N-terminal (RasGEF-N) domain, a DEP domain, a RasGEF domain, a cGMP-binding domain, a GRAM domain (Glucosyltransferases Rab-like GTPase activators and Myotubularins), and a second cGMP-binding domain. In this study, the authors proposed that the MEKK activity of GbpC is intramolecularly regulated by its upstream Ras domain, and that the MEKK activity of GbpC depends on the cGMP transmitted from the cGMP-binding domains through the RasGEF and Ras domains, all occurring within the same protein (Figure 1). This Ras domain is unique with respect to the other canonical Ras-family GTPases, in that both its upstream regulator and its downstream effector kinase are contained within the same polypeptide chain.

Additionally, P.J.M. Van Haastert’s group discovered the region between the Roc and the kinase domain COR (C-terminus of Roc), and called proteins consisting of Roc and COR “Roco proteins” for their unique structures [1]. The group used the amino acid sequence encompassing the Roc-COR tandem domain of GbpC to search for other Roco proteins from various databases. They found Roco proteins in prokaryotes, *Dictyostelium*, plants, and animals, each consisting of a juxtaposition of the Roc and the COR domains; there are no proteins containing either Roc or COR domain alone. Based on their domain architecture, the identified Roco proteins are categorized into three groups. The largest group is found in *Dictyostelium* and metazoan, which is characterized by the central LRR-Roc-COR-Kinase tandem domains (Figure 1). In these proteins, the central architecture comprises the Roc-COR tandem domains, which are always preceded by a leucine-rich repeat domain (LRR) and followed by a kinase domain belonging to the MAPKKK subfamily. The N- and C-terminus flanking the Roc-COR domain consists of remarkably diverse structures, including WD-40-like beta-propeller repeats (WD40 and Kelch motif), and domains which interact with small GTPases (RasGEF, RhoGEF, and RhoGAP) [1] (Figure 1). The second group of Roco proteins is characterized by lacking of the kinase domain is found in mammals (MASL1), the plant Arabidopsis, and prokaryotes [3] (Figure 1). The proteins from this group only contain the central domain architecture, lacking a kinase domain. The Death-associated protein kinase (DAPK1) represent the third group of Roco proteins. DAPK1 is characterized by its death domain, which is found in proteins with apoptotic functions [4,5,6]. The central domains architecture of DAPK is followed by the death domain, whereas the kinase domain is located in the N-terminus, and is the only one that lacks an LRR domain in the group. DAPK1 was first identified in the 1990s [7] as a serine/threonine kinase that is a positive mediator of programmed cell death induced by IFN-γ. 

Although the Roco proteins, such as DAPK1 and a few from *Dictyostelium* and prokaryotes described above, have been studied for some time [1,8], they were not widely studied, and the features of Roc-COR had not been well characterized until two research groups linking mutations in the gene encoding leucine-rich repeat kinase 2 (LRRK2), a human Roco protein, with autosomal dominant Parkinson’s disease (PD) in 2004 [9,10]. Since then, there has been intense interest and research activity in the quest to understand the details of Roco proteins. Due to the association of LRRK2 with both familial and sporadic PD, it has become the most intensively-studied protein among the Roco family. Abnormally high kinase activity of LRRK2 is associated with PD pathogenesis; therefore, the majority of studies have been mainly focused on understanding the role of the kinase activity in relation to the biological function of LRRK2 and in disease. A unique feature of LRRK2 is that it catalyses two distinct biochemical activities, phosphotransfer via its kinase domain and GTP hydrolysis via its Roc domain [11,12]. Although the Roc domains of Roco proteins are annotated as belonging to the small Ras GTPase superfamily, P.J.M. Van Haastert’s group utilized Cluster Analysis for 21 Roc domains and 34 other small GTPases to reveal that the Roc domains were clearly distinguished from the other groups of Ras/GTPase proteins [1,13]. This can explain why the Roc domain of DAPK1 had remained unidentified for decades, despite having been well-studied since the 1990s [7]. In recent years, there has been increasing interests in understanding the role of the GTPase activity of Roco proteins in an effort to understand the pathogenesis mechanisms LRRK2 in PD [8,14]. In this review, we will focus on discussing the potentially unique mechanism of action of the Roc domain compared to the classic Ras/GTPase, and the roles of GTPase activity in the functioning of Roco proteins and in disease.

## 2. Biological Functions of Roco Proteins 

The Roco family of proteins have been identified in a wide phylogenetic range, from bacteria to humans [1]. This family of proteins has drawn intense interest because it has been linked with human disease. For example, mutations in LRRK2 have been associated with PD [9,10], and DAPK1 has been associated with cancer [6,7]. The diversity of domains flanking either side of the central core GTPase and kinase domains, including a variety of regulatory and protein-protein interaction domains, indicate that these proteins have diverse functions, and that the Roc domains appear to serve as the molecular switches that regulate their activities.

### 2.1. GbpC and Pats1 in D. Discoideum

**Figure 2:**
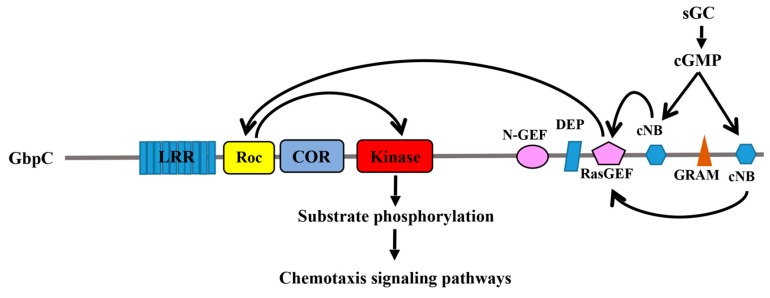
Schematic depiction of the regulatory event that occurs in GbpC. cGMP binding results in the activation of Roc, which in turns activate the kinase domain.

Some of the most studied Roco proteins have been found from *D. discoideum*. Although 11 Roco genes have been identified in *D*. *discoideum* so far [1], GbpC and Pats1 (protein associated with transduction of signal 1) have been the most thoroughly studied. GbpC plays an important role in chemotaxis in slime mold [15,16], a process that involves the phosphorylation of myosin II and its assembly in the cytoskeleton, which is regulated by cGMP binding [16]. In addition to the Roc-COR tandem domain and kinase domain, GbpC has a peculiar C-terminal region that contains a Ras guanine exchange factor (Ras-GEF) domain, a GRAM domain, and 2 cGMP-binding domains. The binding of cGMP to GpbC induces an intramolecular activation mechanism by sequentially activating the Ras-GEF, Roc, and kinase domains [17] (Figure 2). GbpC translocation is induced by cAMP and depends on its GRAM domain to associate with the cellular membrane and cell cortex [18]. At the membrane and cell cortex, GbpC would phosphorylate its substrates via its kinase domain, which in turn is regulated by the upstream signals from Roc [18]. It appears that the Roc domain of GbpC functions by undergoing a G-protein cycle that is reminiscent of the canonical G-proteins, except that the upstream regulator and the downstream effector happen to reside in the same polypeptide chain. It is still unclear whether or not these interesting findings are inferable to all other Roco family proteins.

Pats1 was first identified as a novel gene linked to the cytokinesis by using a phenotype mutagenesis screen [19]. In addition to the GTP-binding domain and kinase domain, Jonathan C. Abysalh and colleagues also identified a protein tyrosine phosphatase domain in the N-terminus of Pats1 (Figure 1). Overexpression of the kinase domain of Pats1 resulted in a severe cytokinesis defect in attached culture cells, and knockout of the Pats1 gene also caused a cytokinesis deficit phenotype [19]. Overexpression of the kinase domain alone was sufficient to rescue the cytokinesis deficit in the Pats1-null cell. In this study, the results suggested that Pats1 has an important role in cytokinesis, but the details of the mechanism involved remained unclear. The GTP binding domain of Pats1 shares sequence homology with both Rho and Ras small GTPases [19]; however, its GTPase activity and its GTP binding kinetics have not been fully characterized. Thus, the role of the GTP binding domain in Pats1-regulated cytokinesis in cell division remains unclear. Interestingly, Pats1 consists of a Roc-COR domain and a kinase domain, but it lacks other regulatory domains that are found in GbpC, such as RasGEF, thus suggesting that Pats1 might require a yet unknown extramolecular regulatory protein to modulate its GTPase activity. 

### 2.2. Human Roco Proteins: DAPK1, LRRK1/2, MASL1

The four human Roco proteins identified so far comprise DAPK1, LRRK1 (leucine-rich repeats kinase1), LRRK2 (leucine-rich repeats kinase 2), and MASL1 (malignant fibrous histiocytoma amplified sequence 1). A common feature among these four proteins is that they all contain a central Roc-COR domain characteristic of Roco proteins; however, the variety of the different surrounding domains illustrates the diverse functions of these proteins. The smallest of the group, MASL1, is composed of only three domains, an LRR domain followed by the Roc-COR tandem domains. It lacks a kinase domain, thereby leaving GTPase as its sole catalytic activity, indicating that most likely the Roc domain is the main functional domain to carry out its cellular functions [8,14]. DAPK1 consists of a kinase domain at the N-terminus followed by an ankyrin repeat (ANK), then the central Roc-COR domain, which in turn is followed by its death domain (which is a protein-protein interaction mediating domain that exists in many apoptosis-promoting proteins) [20]. LRRK2 and LRRK1 are closely related in vertebrates [21]. However, LRRK1 has rarely been reported to be associated with a human disease except that one family was found to carry LRRK1 mutations linked to the osteosclerotic metaphyseal dysplasia (OSMD) [22], while LRRK2 has been strongly associated with PD, Alzheimer’s disease (AD), and immune disorders [23,24]. As such, LRRK2 has been an important potential drug target for PD.

DAPK1 was first discovered as a protein involved in the process of cell death [7], and it was subsequently determined to be a positive regulator of both apoptotic and type-II autophagic cell death [7,25]. DAPK1 might also function as a tumor suppressor by triggering apoptosis. Mutations leading to a reduced expression of DAPK1 have been associated with inheritable predisposition to chronic lymphocytic leukemia [26]. Additionally, DAPK1 may also be involved in neuronal cell death, such as epilepsy and Alzheimer’s disease [27]. The Roc domain negatively regulates DAPK1 activity through binding GTP to the P-loop motif of its Roc domain. The Roc domain of DAPK1 has also been implicated in mediating cellular functions through the formation of a DAPK1 homodimer, as well as by interacting with other Roco family proteins and cytoskeleton, including LRRK2 protein [25,28,29]. 

The biological function of MASL1 is the least understood among the four human Roco proteins. MASL1 was originally identified as a novel gene which was amplified from a malignant fibrous histiocytoma, implying that MASL1 was involved in cell fate and division [30]. Later cellular studies of MASL1 suggested that it plays a role in the regulation of erythroid differentiation of CD34 (+) cells and necrotic cell death through the ERK pathway [31]. The Roc domain of MASL1 has been demonstrated to physically interact with Raf1, which is upstream of ERK signal cascade [31]. Because MASL1 lacks a kinase domain, its Roc domain acts on extramolecular effectors similar to the classic Ras/small GTPase family. 

LRRK1 is closely related to LRRK2, and it was believed that they derived from the same ancient gene via DNA duplication [32]. Both LRRK1 and LRRK2 are expressed widely in human tissues and organs, including the brain. Although they share a very similar multi-domains architecture, mutations in LRRK2 are strongly associated with familial and sporadic PD, whereas mutations in LRRK1 are found in a family with OSMD [22]. Recent findings from Weirong R. Xing’s group suggest that LRRK1 regulates actin assembly in mature osteoclasts by phosphorylating l-plastin [32,33]. 

Among the human Roco proteins, LRRK2 has been the most studied. Human genetics has linked LRRK2 to PD, Crohn’s disease, multibacillary leprosy, and cancer [34]. LRRK2 has been implicated in a wide range of cellular processes including mitochondrial maintenance, synaptic vesicle cycling, autophagy, lysosomal biology, cytoskeletal regulation, neurite outgrowth regulation, and translational control [35,36]. Mice having LRRK2 knocked out showed kidney, lung, and liver abnormalities, including the accumulation of vesicles within cells and alterations in markers for autophagy [37,38]. A more recent study showed that alteration in LRRK2 could also result in defective chaperone-mediated autophagy (CMA) [39]. LRRK2 has been shown to interact with many cytoskeletal proteins, including β-tubulin [40], actin [41] and moesin [42], thus suggesting that LRRK2 might play a role in the control of cytoskeletal remodeling and regulating neurite outgrowth [43,44,45]. An increasing number of studies are implicating LRRK2 functioning at membranous structures [46,47]. In 2014, Mark Cookson’s group performed a chip-based LRRK2 interaction screening assay and found a number of interactors known to be involved in the vesicular recycling process, including Rab7L1, which is also involved in intracellular protein sorting [48,49]. Further investigation by Dario Alessi’s group showed that Rab7L1 recruits LRRK2 to the trans-Golgi network, and that this interaction activates the kinase activity of LRRK2 [50]. Using a phosphoproteomics approach, the same team also identified a subset of Rab GTPases as potential substrates of LRRK2 [51], then they used systematic proteomic analysis to identify ten specific Rabs that were endogenously phosphorylated by LRRK2 [52], thereby identifying a potential role for LRRK2 in ciliogenesis. Subsequently, the same team in collaboration with Susan Pfeffer’s group showed that LRRK2 blocks primary cilium formation through the Sonic hedgehog signaling pathway [53]. 

Taken together, it appears that LRRK2 plays an important role in a number of different cellular processes involving membranous structures; however, its precise function(s) and mechanism in disease pathogenesis remain elusive.

## 3. Biochemical Activity of Roc and Its Regulatory Mechanisms

The typical small G-proteins function as molecular switches by cycling between the GDP-bound inactive and GTP-bound active conformations. This biochemical cycle is composed of a binary process; the GDP-bound inactive state is switched to the activated state upon GTP binding, and reciprocally, the GTP-bound active state is switched off by the intrinsic GTP hydrolysis activity of G-protein. These changes are intricately controlled by GEFs, which promote the exchange of GDP with GTP, and by GAPs, which accelerate the biochemical hydrolysis of GTP. Utilizing this highly-conserved functional G-protein fold, their different subcellular localization and specific downstream effectors enabled the small G-proteins to regulate a diverse set of biological processes in the cell. The small G-proteins in the Roco family of proteins are unique, in that they are intramolecularly-linked with a COR domain, and their nucleotide exchange process might not require GEFs or GAPs. However, the molecular details of their mechanism of action remain to be elucidated. 

### 3.1. Roco Proteins are Functional GTPases

G-proteins are characterized by two main functions: guanosine nucleotides (GDP and GTP) binding and GTP hydrolysis. The guanosine nucleotide-binding property of the Roco proteins has been widely reported for proteins from prokaryotic to human [25,28,54]. Wouter N. van Egmond and colleagues used GTP-agarose pull down to assay for the GTP binding ability of *D*. *discoideum* GbpC, and they demonstrated that the nucleotide exchange process of Roc domain of Gbpc was specifically activated by its C-terminus GbpC-RasGEF domain [17]. Katja Gotthardt and colleagues quantified the nucleotide binding ability of *C. tepidum* Roco in vitro by using purified recombinant Roc-COR domain, which showed that Roco has the low binding affinity for both GDP and GTP (in the micromolar range) [55]. Guanosine nucleotides binding of the human Roco proteins has been well studied. Rodrigo Carlessi and colleagues showed that an excess of free GTP could competitively deplete the pulled-down DAPK1 from GTP-agarose and that the artificial mutation T701N in the P-loop motif of Roc domain, which would disrupt GTP binding, significantly increasing the kinase activity of DAPK1 compared to that of the WT [28]. Sybille Dihanich and coworkers measured GTP binding for MASL1 using GTP-agarose pull-down assay in mammalian cells and showed that GTP binding was necessary for the complex formation of MASL1 in cells [56]. There is also an abundance of evidence showing human LRRK1 and LRRK2 binding with GTP and GDP [54,57]. Both LRRK2 full-length and its isolated Roc domain or Roc-COR tandem domain have been reported to selectively bind to GDP and GTP with similar affinity [12,54,55], and that mutations in the nucleotide-binding P-loop, such as K1347A or T1348N, resulted in a reduction of GTP/GDP binding [58]. 

**Figure 3:**
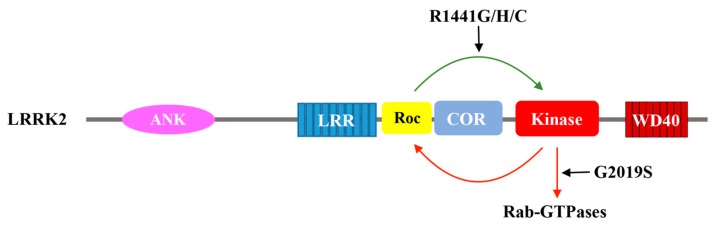
Schematic depiction of the catalytic activity of LRRK2. GTP binding to Roc activates the kinase domain, which in turn phosphorylates downstream substrates and potentially feedback to the Roc domain via phosphorylation. The Parkinson’s disease-associated mutation G2019S aberrantly over-activate the kinase domain. A set of Parkinson’s disease-associated mutations in the Roc domain (R1441G/H/C) also activate LRRK2 kinase activity.

Similarly, the GTP hydrolysis activity of the Roc domains of the Roco proteins has been well characterized, including DAPK1 [28], LRRK2 [12], and the *Chlorobium tepidum* Roco [55]. Among them, LRRK2 has garnered the most attention. Several groups have demonstrated that LRRK2 is able to hydrolyze GTP using radioactively labeled guanine nucleotide to assess its intrinsic GTPase activity in vitro [54], and this activity is disrupted when the key residues in the P-loop required for nucleotide described above are mutated. Truncation constructs consisting of just the Roc domain or the Roc-COR tandem domain retains LRRK2 GTPase activity; however, the activity is significantly lower than that of the full-length protein, indicating that the GTPase activity may be intramolecularly modulated via interaction with other domains (Figure 3) [12,55,59]. Although the mechanism of action of the Roc G-protein remains unclear, it might be different from that of the typical G-proteins. For example, LRRK2 carrying the mutation R1398L resulted in a protein that possesses higher GTPase activity, while the equivalent mutation in Ras would abolish GTPase activity and trap it in a persistently active form [58,60]. A dual substitution mutant, combining the R1398L with a T1343V, in P-loop has created an active form of LRRK2 with impaired GTPase activity [58]. These artificial mutations confirm the GTPase activity of the Roc domain of LRRK2, and suggest that it might function a little differently than the typical G-proteins.

### 3.2. Regulatory Mechanisms of Roc Activity

As mentioned above, the Roc domain of GbpC from *D*. *Discoideum* is uniquely regulated by its RasGEF domain in the same polypeptide chain. A number of studies have set out to identify the GEFs and GAPs for LRRK2. One of the potential GEFs identified for LRRK2 is ArhGEF, which is a GEF of CDC42 and Rac1, and its interaction with LRRK2 appears to increase GTPase activity in cells and mouse brain [61,62]. ArhGEF binding to LRRK2 might be dependent on the phosphorylation state of LRRK2, which is stimulated by CK1α [63]. Interestingly, ArhGEF can also be phosphorylated by LRRK2 in vitro [62]; however, it is unclear how ArhGEF affects the kinase activity of LRRK2 and how its phosphorylation of ArhGEF affect the activity of the GEF. It has been shown that the kinase activity of LRRK2 is toxic in yeast and a genetic screen for suppressors of this toxicity has found a GAP, GCS1, as a suppressor of LRRK2-induced toxicity [60]. The mammalian homologue of GCS1, ArfGAP1, has been tested for its interaction with LRRK2 in human cells and in rodent brains, and in vitro assays showed that ArfGAP1 causes an approximately 2–3 fold acceleration of the GTPase activity of LRRK2 [64,65]. Surprisingly, ArfGAP1 is found to interact with the N-terminus and C-terminus portions of LRRK2 rather than at the expected catalytic core region where the Roc domain resides [64,65]. If ArfGAP1 is indeed a GAP for Roc, then it would indicate that the GAP activating mechanism of Roc is more complex and different than that of the canonical small GTPases. Another potential GAP for LRRK2 is RGS2, which was originally identified in *C. elegans* as a modulator of LRRK2 [66]. RGS2 is subsequently shown to interact with LRRK2, and in so doing, increases its GTPase activity leading to a reduction in kinase activity *in vitro* [66]. It is unclear how ArfGAP1 and RGS2 both activate GTP hydrolysis of LRRK2 while showing the opposite effect on its kinase activity and neuronal toxicity. It remains to be determined whether or not ArfGAP1 or RGS2 are authentic physiological GAPs of LRRK2.

Interestingly, biochemical characterization of the LRRK2 protein reveals that the guanosine nucleotide affinity is much weaker than that of the canonical small GTPases [12,55]. Generally, the nucleotide affinity of Ras family of proteins is in the range from picomolar to nanomolar, whereas the nucleotide affinity of LRRK2 is in the micromolar range. The low affinity or nucleotides would enable nucleotide exchanges to occur in LRRK2 in the absence of GEFs. There is a precedent for a GEF- and GAP-independent G-protein activation mechanism. Raphael Gasper and colleagues classified a group of G proteins that are regulated by dimerization as GADs (G-proteins activated by nucleotide-dependent dimerization), including the SRP (signal recognition particle), the dynamins, and the septins [67]. Indeed, LRRK2 has been proposed as a GAD G-protein based on the structural study of prokaryotic *C. tepidum* Roco protein model [55]. The basic mechanistic feature that distinguishes GADs from the typical small G-proteins is that GADs do not require GEFs and GAPs for nucleotide exchange and GTPase activation, but instead, nucleotide exchange is dependent on the local concentration of the nucleotides, and GTPase activation is conferred upon dimerization [67]. The notion that LRRK2 is a GAD G-protein has been inferred from the studies of the *C. tepidum* Roco. A Roc-COR contiguous construct of the CtRoco has been crystallized as constitutive homodimer stabilized by the interactions between the two COR domains. Based on the structure, it was proposed that the dimerization mediated by the two COR domains would position the Roc domains in close proximity to each other, thereby exchanging an Arginine residue that would complement and constitute a productive catalytic site [55]. Katja Gotthardt and colleagues suggested that the structure of the prokaryotic CtRoc-COR construct has implications for LRRK2, although the key active-site residues are not conserved [55]. Even so, there is evidence consistent with LRRK2 being a GAD G-protein. For example, LRRK2 has been reported to predominantly exist as a homodimer in cells [68,69], its COR domain dimeric interface is the most highly-conserved region among Roco proteins [70], the isolated COR domains of LRRK2 interact with each other [55,59], and the familial mutations (R1441C/G/H) disrupt dimerization and reduce GTPase activity [71,72]. However, several lines of evidence have shown that the isolated Roc domain of LRRK2 is catalytically active in its monomeric state in solution [12,73], demonstrating that dimerization is not essential for its GTP hydrolysis activity (which is inconsistent with the GADs model). Of note is that the catalytic arginine residue R543 of the ortholog *C. tepidum* Roc-COR does not exist in human LRRK2; therefore, it is still unclear whether the mechanism of action of CtRoco is the same as that of the human LRRK2. Another observation of LRRK2 that is inconsistent with the GAD model is that the binding of nucleotides does not regulate LRRK2 dimerization in cells. On the other hand, the human DAPK1 forms a homodimer in cells through interactions in the Roc and kinase domain instead of the COR domain.

More recently, Egon Deyaert and colleagues proposed that the GTPase catalytic activity of the *C. tepidum* Roco protein is regulated by the conformational changes between dimeric and monomeric states during the process of GTP turnover [74]. In contrast to the GAD model mentioned above, the current dimer-monomer cycle model posits that GTP binding induces monomerization, and that GTP hydrolysis occurs in the monomeric form of the enzyme. LRRK2 has been observed to cycle between a dimeric kinase-active form and a monomeric kinase-inactive form in vivo [69,75]. The studies demonstrated that stabilization of either the dimeric or monomeric state of LRRK2 led to decreased GTPase activity [74]. 

Additionally, emerging evidence has indicated that the intramolecular kinase activity might reciprocally regulate the GTPase activity [73,76]. For example, autophosphorylation sites mapping of LRRK2 using mass spectrometry has found that many of these sites are clustered in the Roc domain, such as T1343, T1348, S1403, T1404, T1410, and T1503 [77,78,79]. Interestingly, these residues map primarily to the P-loop motif (which involves in nucleotide binding) and the switch regions (which mediate GTP binding or GTP hydrolysis) of Roc, suggesting that autophosphorylation at the Roc domain could potentially affect nucleotide binding and/or GTP hydrolysis. It has been reported that the phosphorylation of residue T1503 may regulate GTP binding and kinase activity of LRRK2 [11]. Another study showed that the phosphorylation of residues in the Roc domain increases the rate of GTP hydrolysis [73]. In addition to the intrinsic kinase activity, an extrinsic kinase PKA has also been shown to phosphorylate residue S1444 of LRRK2, where the phospho-binding protein 14-3-3 has been shown to dock, and in turn, 14-3-3 binding causes a decrease in the kinase activity of LRRK2 [80].

In addition to the cluster of sites located in the Roc domain mentioned above, autophosphorylation sites are also found in the region between the ANK domain and LRR domain, including S910, S935, S955, and S973 [81]. Since the phosphorylation of LRRK2 is a potential regulation mechanism of LRRK2, then its dephosphorylation might also a play a role. Indeed, several pathogenic mutants in LRRK2 have been shown to have a reduced phosphorylation level at the above ANK-LRR inter-domain phosphosites [82]. Moreover, inhibition of LRRK2 also resulted in a reduction of phosphorylation at these sites, thus lending support to the notion that the phosphorylation and dephosphorylation of LRRK2 are potentially a regulatory mechanism of LRRK2, as thoroughly reviewed by Jean-Marc Taymans [83]. Two potential LRRK2 phosphatases have been identified in recent years. In 2013, Evy Lobbestael and colleagues showed that the protein phosphatase 1 (PP1) can effectively dephosphorylate LRRK2 at S910, S935, S955, and S973 [82]. More recently, in 2017, Panagiotis Athanasopoulos and colleagues identified protein phosphatase 2A (PP2A) as another potential LRRK2 phosphatase [84]. This study showed that PP2A interacts with the Roc domain of LRRK2, and that knocking down the activity of PP2A aggravated cellular degeneration induced by a pathogenic LRRK2 mutant. The mechanisms of how each phosphosite affect the activity of LRRK2 is unknown, but because LRRK2 is a large mutli-domain and dual enzymatic protein, the different phosphosites might regulate the distinct characteristics. In additional to the conventional effects of phosphorylation on protein-protein interactions and conformational changes, Jing Zhao and colleagues reported recently that LRRK2 dephosphorylation could lead to ubiquitination, thus suggesting that the dephosphorylation of LRRK2 might also regulate its levels in the cell [85].

## 4. The Mechanism of Roc Transmitting Its Signal Downstream

The typical Ras-family GTPases undergo guanine nucleotide-dependent conformational changes that alter their interactions with various effectors in the cellular signaling cascades leading to changes in subcellular localization and/or oligomeric states. However, the Roco-family G-proteins are contiguously fused with a COR domain, and most of them comprise of an intramolecular kinase domain, among others. Given this modular arrangement that consists of a G-protein and a potential effector kinase in the same polypeptide chain, it is tempting to imagine that the Roc domain might interact with its intramolecular kinase domain in a fashion analogous to that of Ras with the MAP kinases. Indeed, in slime mold, the activation of the Roc domain of GbpC with GTP stimulates the activity of its kinase domain, which then goes on phosphorylates its downstream substrates [17,18]. Similar evidence also exists for the human Roco proteins. Daniel Korr and colleagues observed that the kinase activity of LRRK1 could be stimulated by GTP binding [57], and that this kinase activity, which modulates osteoclast function, is regulated by cycling between GTP and GDP-bound Roc domain, which is reminiscent of the canonical Ras-family small GTPases [32,33]. More recently, several lines of evidence demonstrate that the kinase activity of LRRK2 is also regulated in a GTP-dependent manner [86,87,88]. The Roco proteins, lacking an intramolecular kinase domain, appear to transmit their signals extramolecularly, which is similar to the canonical G-proteins. For example, the Roc domain of MASL1, which lacks an intrinsic kinase domain, is also activated by GTP-binding; however, once activated, it regulates the extrinsic kinases in the Raf/MEK/ERK signaling cascade that is involved in the erythropoiesis. Interestingly, in contrast to LRRK1/2 and MASL1, GTP binding to the Roc domain of DAPK1 results in the down-regulation of its kinase activity [17,18]. All this suggests that the mechanisms of action of the Roc G-proteins are more complex than those of typical small G-proteins; however, the mechanism of nucleotide binding-induced conformational changes leading to the toggling of the molecular switch observed in the typical G-protein appear to be conserved in the Roc proteins. 

It is still unclear how the nucleotide-dependent conformational changes in the Roc domain of the Roco proteins result in the activation of their kinase domain. However, several lines of evidence suggest the involvement of dimerization and/or overall conformational changes leading to rearrangement of the domains. For example, Rodrigo Carlessi and colleagues revealed that the Roc domain of DAPK1 mediates its homodimerization as well as its heterodimerization with other kinases, including LRRK2 and ZIPK (ZIP-kinase); however, the mechanistic significance of the dimerization event was unclear at the time [28]. They also showed that deletion of the Roc domain resulted in a more-active DAPK1, indicating that dimerization of the Roc domain is not required for DAPK1 enzyme activity, yet dimerization might be crucial for GTPase activity which directly regulates kinase activity [28]. In contrast, a number of studies have shown that purified full-length LRRK2 forms dimers in solution in vitro and cells [68,89,90]. Saurabh Sen and colleagues observed that LRRK2 isolated from different species were only catalytically active in their dimeric form [68]. Nicholas G. James and colleagues found that the majority of cytoplasmic LRRK2 is monomeric, and that the LRRK2 oligomers, including dimers, tetramers, or higher-order oligomers, are associated with the plasma membrane or the intracellular membranous structures [49,91]. Other studies have shown that the membrane-associated LRRK2 is dimeric and is more catalytically active than the cytosolic monomeric conformation [69]. As such, it appears that guanine nucleotide-binding drives Roc conformational changes that, in turn, alter the conformation and/or oligomeric states of the Roco proteins, ultimately leading to altered protein-protein interactions and/or subcellular localization. However, the defining feature that distinguishes Roc from all other G-proteins is the juxtaposition of Roc and COR. Roc always co-exists contiguously with a COR domain, suggesting that they likely function as a unit; however, how the two domains work together is still a mystery. Understanding the interplay between Roc and COR is essential for understanding the mechanism of action of this unique class of G-proteins. 

## 5. Conclusions

The Roco family of G-proteins is the most recently characterized and least understood of the Ras-like small G-protein family. It is a class of G-proteins in which the G-domain functions within a multi-domain protein and is characteristically conjoined with a COR domain. Understanding the details of the functioning of the Roco proteins will have important applications. For example, LRRK2 is currently the most promising drug target for Parkinson’s disease, and it is being intensively investigated for that purpose. Because the aberrant kinase activity of LRRK2 is associated with disease pathogenesis, most efforts in drug discovery have been focused on inhibiting its kinase domain. This has yielded a number of highly specific and potential inhibitors of LRRK2 kinase activity [92,93,94]. However, inhibition of LRRK2 kinase activity has led to detrimental side-effects in animal models [37,38,95,96]. Still, researchers in the field are working to modulate LRRK2 kinase activity to achieve therapeutic doses and minimize side-effects.

The unique structure comprising of the Roc-COR domains might provide an alternative venue for therapeutic development. This is an attractive strategy because, in addition to the unique structure that might offer specific drug binding sites, it is an intrinsic modulator of the kinase domain; thus, modulating the modulator might offer a tunable regulation of LRRK2 kinase activity. To achieve this, we will need to understand the biochemical and structural details of the Roc-COR domains. The bottleneck in these endeavors has been obtaining sufficient amounts of correctly folded proteins suitable for the biochemical and biophysical studies. Even so, there are ongoing efforts to achieve precisely that.

## Abbreviations

GEFs	Guanine nucleotide exchange factors
GAPs	GTPase-activating proteins
Roc	Ras of complex proteins
GbpC	cGMP binding protein C
LRR	Leucine-rich repeat
COR	C-terminal of Roc
LRRK1/2	Leucine-rich repeat kinase 1/2
MASL1	Malignant fibrous histiocytoma amplified sequence 1 (MFHAS1)
DAPK1	Death-associated protein kinase 1
GRAM	Glucosyltransferases Rab-like GTPase activators and Myotubularins
PD	Parkinson’s disease
AD	Alzheimer’s disease
OSMD	Osteosclerotic metaphyseal dysplasia
CMA	Chaperone-mediated autophagy
GADs	G-proteins activated by nucleotide-dependent dimerization
CK1	Casein kinase 1 alpha
PP1	Protein phosphatase 1
PP2A	Protein phosphatase 2A
